# Clinical Lectures upon Surgical Cases in the Buffalo Hospital of the Sisters of Charity

**Published:** 1871-11

**Authors:** J. F. Miner


ART. III.—*Clinical Lectures upon Surgical Cases in the Buffalo Hospital of the Sisters of Charity.* By PROF. J. F. MINER.

*Reported by Louis Brecht, member of the class of* 1871-72.

CASE V.—*Caries of the Bones of the Foot.* D. Gleason, aged 21 years, admitted into the Hospital about three months ago, for the treatment of an abscess on his right foot. At the time of his admission he was able to walk with slight difficulty, but soon after became unable to do so and was confined to his bed. You will now observe a large unhealthy opening which has the appearance of communicating with dead bone. The opening is situated directly above the Scaphoid and internal Cuneiform, and it is possible that these are in a state of decay. A spicula of bone escaped this morning through this opening, but from which bone it came is uncertain. The patient is tired and disgusted with his foot, and has urged me to adopt such measures as would lead to a radical cure. The patient has resolved to submit to any operation except amputation, and I propose removal of whatever bones may be found diseased. Excision of the bones of the foot, is comparatively a new operation, and was not thought of until about thirty years ago.

All cases of caries, involving these bones were formerly treated by amputation. Recently, the subject has received more attention and authors favor the method of exsecting some, or even all of the small bones. It was thought that incisions into joint cavities were unsafe on account of dangers of inflammation, profuse suppuration, absorption, erysipelas, &c. Added to this, it takes a very long time before the foot will get well, while after amputation quicker recovery may be expected. Such cases are sometimes left to the resources of nature; but nature at times is very slow, and the majority of patients have not the means or patience to wait for it. As I introduce my finger through the opening for the purpose of ascertaining the extent of the disease, (the finger being the best probe) I find that not only the Scaphoid and internal Cuneiform are involved but also the head of the astragalus, the middle and external Cuneiform and all require removal. The foot will shorten some, and the cavities caused by the excision of these bones will fill up with a cartilaginous mass which may possibly in time become ossific in greater or less degree. Should the result prove favorable, the operation will indeed be an achievement in surgery, but a favorable result is somewhat doubtful. The patient has a pale and anemic countenance, a weak constitution, and will, perhaps, be unable to bear the exhaustive drain upon the system. The afterdressing will consist of dilute carbolic acid water lotions, which act both as a disinfectant and stimulent to the parts. Good diet and general good care are indispensible.

CASE VI.—*Concussion of the Brain.* Robert Henderson, aged 35 years, a laborer by occupation, fell a few days ago through the hatchway of a vessel, a distance of about sixteen feet. He struck on his forehead, which at the time was supposed by attendants to be fractured. Upon examination on the following morning, I could find no depression or fracture. I found him in an insensible condition, breathing heavily, and it was impossible to arouse him out of this stupified condition. His pulse was weak and frequent, and the pupil normal. The question which presents itself to the surgeon in making his diagnosis in such cases is; "Is there concussion of the brain, or does the patient suffer from compression?" This question is of great importance, and has often puzzled the most experienced surgeons. You cannot, therefore, make yourselves too familiar with the differential diagnosis, and we will here mention some of the symptoms. In concussion we have generally but partial insensibility, breathing not sterterous, pulse weak and intermittent, the voluntary muscles weakened but not paralyzed, and the pupil usually contracted. In compression we have depressed bone or an effusion of blood or serum which presses upon the brain, and renders the patient comatose. The breathing is sterterous, and the pulse often very slow and irregular, while the opposite side of the body to that where compression has taken place is often paralyzed. The patient referred to was soon after the injury brought to the Hospital, and to-day, three days after the accident, we find that his condition has generally improved. He gives some signs of feeling, and when aroused and spoken to, seems to take notice of it, and gives a muttering sound as if attempting to answer. He takes food and drink to some extent. By comparing these symptoms you will at once recognise the condition as probably concussion of the brain. After observing the case and hearing what I have said of the symptoms of concussion and compression, you will at once perceive that the distinction in tipical cases is plain enough, and you will also see that the conditions frequently approach each other so nearly that the surgeon may have to watch the progress of the case before he can make positive diagnosis.

Case VII.—*Spinal Curvature.* The little patient, presented to you this morning, is affected with angular curvature of the spine, and associated with it is quite well marked lateral curve. You observe that the lumbar vertebræ form nearly a complete arch, which is due to pathological changes having taken place in the bodies of these vertebræ. Various theories have been advanced as to the character of these changes. They have been supposed to be scrofulous and tubercular in character—to arise from a peculiar constitution or diathesis, and tubercular deposits have been thought to be in and around the bones. This theory is quite prevalent, and is generally adopted by the majority of the profession. Still I am not prepared to accept this theory at present. Recent observation and investigation go far to disprove this theory. Virchow, and other pathologists have examined the effused products in these so called tubercular deposits formed in and around the spinal curve, and find nothing unlike the products of inflammation. Again, the clinical history of such cases shows almost conclusively that it is not tubercular in character, such cases more often recover. Tubercular disease is almost uniformly fatal. Most of the cases of spinal curve can be traced to fall or injury, and this appears to me much more probably the true cause.

I have had ample opportunity to trace the clinical history of such patients, and have found that they had received injury to which the disease might fairly be attributed. In the treatment of this disease cod liver oil, and all similar remedies have been largely recommended with the view of treating it as a constitutional affection. Cod liver oil may be very proper, but will entirely fail to produce satisfactory results. Hygienic treatment, rest, and a support, made in such manner as to relieve the weight of the body above the curve, will be of much greater value. Indeed, proper support by mechanical means is the chief indication.

